# Multiple Extrapulmonary Tuberculous Abscesses Developed Postpartum in a Non-HIV Patient Under Anti-tuberculosis Chemotherapy

**DOI:** 10.7759/cureus.21395

**Published:** 2022-01-18

**Authors:** Efthymia Papadopoulou, Christina Rampiadou, Evangelos Petsatodis, Diamantis Chloros, Afroditi Boutou

**Affiliations:** 1 Department of Pulmonary Medicine, George Papanikolaou General Hospital, Thessaloniki, GRC; 2 Department of Interventional Radiology, George Papanikolaou General Hospital, Thessaloniki, GRC

**Keywords:** extrapulmonary tuberculosis, abscess, lymhadenopathy, paradoxical reaction, post-partum, immune reconstitution

## Abstract

Although abscess formation constitutes a recognized complication of tuberculous lymphadenitis, the concomitant development of multiple tuberculous abscesses in the course of tuberculous lymphadenitis has rarely been described in the literature among HIV-negative patients under appropriate chemotherapy. Adherence and sensitivity to the administered anti-tuberculosis chemotherapy have to be verified in such patients. We report a case of deteriorating tuberculous lymphadenitis, presenting with the development of multiple extrapulmonary abscesses (cervical, psoas, and retroperitoneal) in an HIV-negative patient who had complied with appropriate anti-tuberculosis chemotherapy for four months. Mycobacterium tuberculosis was the identified pathogen in specimens from the abscesses. Continuation of anti-tuberculosis medications and concurrent administration of antibiotics, along with CT-guided percutaneous drainage of the psoas abscess, resulted in gradual resolution of the patient's lesions. Interestingly, our patient had recent childbirth, indicating a potential association between the immunomodulatory processes during the postpartum period and the development of the so-called paradoxical reaction. Awareness of such complications should be raised, as a timely recognition and subsequent therapeutical treatment are essential for a favorable outcome.

## Introduction

Paradoxical reaction in patients under anti-tuberculosis chemotherapy, presenting with deterioration of tuberculous lesions or even development of new ones, has already been reported in association with tuberculous lymphadenitis, especially in HIV-positive patients [1]. Prior to attributing such deterioration to this immune-related entity, exclusion of other pathological conditions, verification of the patient's adherence to the anti-tuberculosis treatment, along with confirmation of the mycobacterium's sensitivity to the administered chemotherapy, comprise the parameters that have to be principally addressed. We present a case of paradoxical reaction with concomitant development of multiple extrapulmonary tuberculous abscesses in an HIV-negative patient during the postpartum period, four months after anti-tuberculosis treatment initiation, which was treated with a combination of drug therapy and percutaneous drainage. To the authors' knowledge, such cases have scarcely been reported in the literature. The potential association between the immunomodulatory processes during the postpartum period and the development of paradoxical reaction is highlighted by our patient's recent childbirth.

This case report was previously presented as a meeting abstract at the 4th Panhellenic Conference of Chest Diseases on May 28, 2021.

## Case presentation

A 27-year-old female Afghan immigrant presented to the emergency department complaining of progressively worsening pain in the left inguinal region for the past two weeks. She was already under chemotherapy for the past four months (rifampin, isoniazid, pyrazinamide, and ethambutol for two months and rifampin, isoniazid, and ethambutol for another two) with a diagnosis of tuberculous lymphadenitis. Lymphadenitis had developed during the past year with manifestations of fatigue, weight loss (14 kilograms within a period of three months prior to treatment initiation), and painful swelling of the cervical lymph nodes, especially in the left supraclavicular and lateral cervical region. Noteworthy was also the fact that she had had her third childbirth eight months ago.

**Figure 1:**
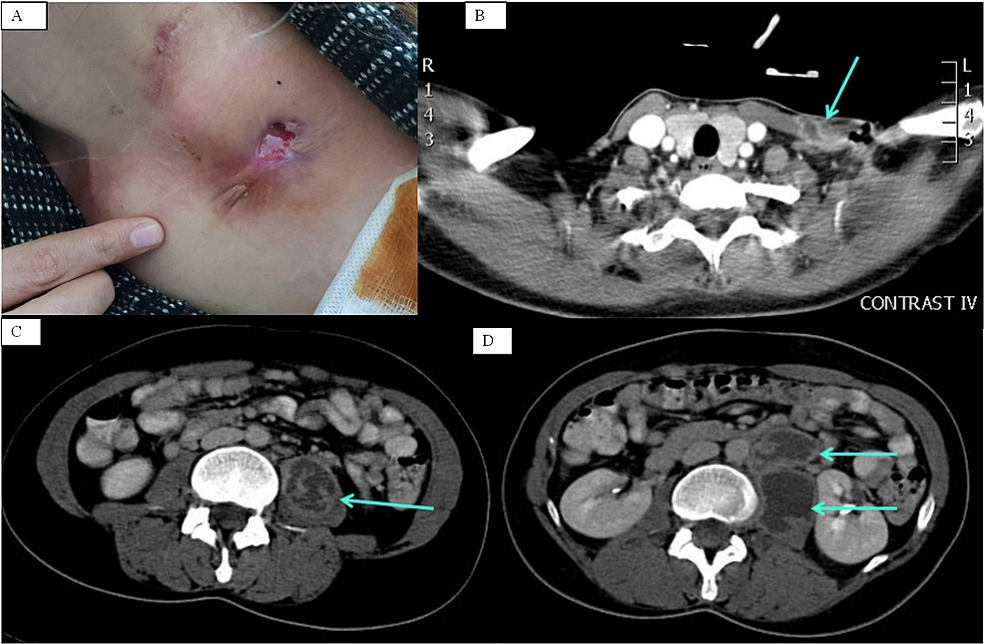
Multiple extrapulmonary tuberculous abscesses A: spontaneous rupture and fistulae in cervical tuberculous abscess; B: left-sided cervical abscess surrounded by swollen lymph nodes; C: left-sided psoas abscess containing diaphragms; D: left-sided psoas and retroperitoneal abscesses.

On physical examination, she had a body mass index of 21.5 kg/m^2^; she was afebrile and hemodynamically stable, with normal breath sounds. Three apparent swollen lymph nodes were observed in the cervical area, one of which seemed to have developed fistulae (Figure 1A). There was also tenderness upon palpation of the left inguinal region. Further assessment of this area was acquired through an abdominal ultrasound, which revealed a hypoechoic lesion of 8.5x3 centimeters within the left psoas muscle. A computed tomography (CT) scan of the abdomen confirmed this pathological finding depicting a cavitating abscess that was located within the left psoas muscle and contained diaphragms (Figure 1C). Moreover, another cavitating abscess of 3x2 centimeters was located in the retroperitoneal space (Figure 1D). In order to investigate the pathological lymph nodes in the cervical region, a CT scan of the area was performed, which revealed another abscess in the left supraclavicular region, surrounded by swollen supraclavicular lymph nodes (Figure 1B). Chest X-Ray demonstrated no signs of active pulmonary tuberculosis, whereas a chest CT scan featured calcified nodules bilaterally, along with calcified lymph nodes and areas of atelectasis.

Due to the development of multiple abscesses despite treatment with anti-tuberculosis chemotherapy for four months, suspicion of human immunodeficiency virus (HIV) infection was raised, but the subsequent examination yielded a negative result. Blood laboratory tests revealed mild anemia (hemoglobin 11.2g/dL), as well as slightly elevated values of C-reactive protein (3.02 mg/dl) and erythrocyte sedimentation rate (72 mm) with a negative procalcitonin and normal leukocyte count and type. The patient did not appear to be malnourished, and serum albumin amounted to 4.25 gr/dl, whereas total protein to 7.68 gr/dl. Mantoux test was measured positive at 18mm. Ziehl-Neelsen stain as well as Löwenstein and mycobacterial growth indicator tube (MGIT) culture tests were performed in fluid specimens of both the psoas and the supraclavicular abscesses, but proved negative, as did the aerobic, anaerobic and fungal cultures. Upon further molecular testing with Xpert® mycobacterium tuberculosis/rifampin (MTB/RIF) assay, mycobacterium tuberculosis sensitive to rifampin was identified in both of the aforementioned specimens.

**Figure 2:**
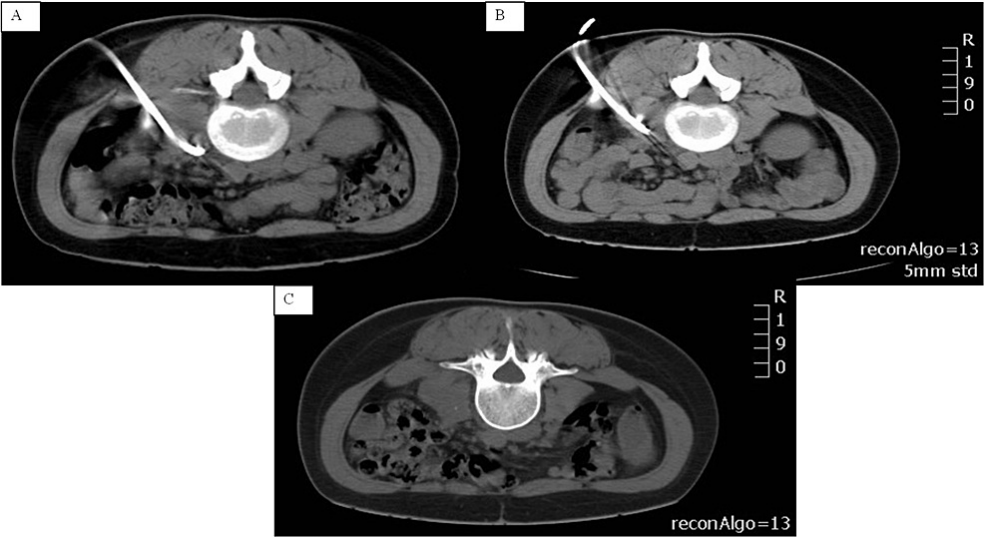
Percutaneous drainage of the psoas abscess under radiological guidance A: CT-guided pigtail catheter insertion for the drainage of the psoas abscess; B: pigtail catheter removal after complete drainage of the purulent fluid; C: the significant resolution of the psoas abscess achieved through percutaneous drainage.

During hospitalization, the patient continued anti-tuberculosis treatment with rifampin, isoniazid, and ethambutol. Furthermore, amoxicillin-clavulanic acid was administered for 10 days in order to impede and treat potential contamination of the abscesses. Therapeutical drainage of the psoas abscess was achieved by insertion of a pigtail catheter under CT guidance (Figure 2, A-C). The fluid had a purulent character and amounted to 200cc.

The patient was discharged after a hospitalization period of three weeks, during which a gradual improvement in her clinical condition was noted. Follow-up went on for one year with a monthly evaluation of the patient's clinical condition and laboratory findings. During the following three months, the secretion of pus from the supraclavicular abscess gradually subsided. She continued anti-tuberculosis chemotherapy for a total of nine months without further complications.

## Discussion

The development of multiple extrapulmonary tuberculous abscesses in a patient already under treatment for tuberculous cervical lymphadenitis is intriguing. Before progression of the disease is hypothesized, other pathological conditions, including immunosuppression secondary to HIV, are to be excluded. Additionally, appropriate medication compliance should not be overlooked, whereas potential development of resistance to the anti-tuberculosis medications also has to be ruled out. In the case we report, all these potential causes were excluded. 

Abscess development is not an infrequent complication of tuberculous lymphadenitis since it has been reported at a percentage of 5-22% [2], but documented at even higher rates, up to 34.5%, in a study conducted at a referral hospital [3]. As a complication, the formation of cutaneous fistulae or even spontaneous rupture of the abscess may occur in patients under treatment [3] as in our patient, who presented with a cervical abscess and fistulae while under appropriate anti-tuberculosis chemotherapy. Regarding the therapeutical approach of such patients, it has been proposed that surgical excision expedites recovery and potentiates the efficacy of medication [4], but, according to the Infectious Disease Society of America (IDSA), such treatment should be considered in limited cases of drug-resistance or persistent deterioration [5]. Our patient demonstrated sensitivity to the medications which she had already been receiving and, therefore, conservative management with the continuation of anti-tuberculosis treatment, along with a meticulous dressing of the cervical abscess, in order to avoid contamination, was followed, resulting in gradual clinical improvement with the decline in pus excretion and resolution of the cervical abscess.

Apart from the cervical region, two additional tuberculous abscesses, one located in the retroperitoneal space and another one within the psoas muscle, had developed. First mentioned in 1881 by Herman Mynter [6], non-tuberculous psoas muscle abscess has been amply reported ever since. The psoas' abundant blood supply, owing to its type 2 muscle fibers and high myoglobin content, predisposes to hematogenous spread to this muscle [7]. Usually, in cases of psoas abscesses, *Staphylococcus aureus* is the identified pathogen, whereas enteric bacteria such as Streptococcus species (4.9%) and *Escherichia coli* (2.8%) [7] have also been inculpated. Mycobacterial psoas abscess constitutes a recognized complication of concomitant spondylodiscitis (Pott's disease) [8]. However, reports are only scarce in cases sans spinal tuberculosis, as in our patient. A wide variety of pathological conditions comprises the differential diagnosis [7], since manifestations are often non-specific, with the classical clinical triad -namely fever, back pain, and lower extremity weaknesses - presenting in less than one-third of the cases [9]. In our patient, the gradual emergence of pain, with no detectable fever, was compatible with the insidious accumulation of pus within the psoas muscle.

The treatment of a tuberculous psoas abscess includes the administration of appropriate anti-tuberculosis chemotherapy, combined with fluid drainage, which may be either surgical or percutaneous. The latter is achieved through catheter insertion under radiological guidance and is therefore preferred in cases of uniloculated abscesses [10]. However, upon failure of percutaneous drainage and in cases of multiloculated abscesses, concomitant abdominal pathology requiring surgical intervention, or persistent neurological deficits, open surgical drainage should be performed [11]. In our patient, percutaneous drainage of the purulent fluid led to amelioration of pain in the inguinal region, as well as improvement in her overall clinical condition. Tuberculosis-induced immunosuppression is considered to constitute a predisposing factor for concomitant bacterial infections, which have already been observed in patients with tuberculous abscesses [12]. Therefore, concurrent administration of anti-tuberculosis medications and antibiotics against staphylococcal species, as well as enteric bacteria, is to be timely initiated in such cases and properly adjusted to the results of the fluid culture.

The development of multiple abscesses in our patient may be explained in view of the so-called paradoxical reaction (PR). Such cases have already been reported in the literature, and it has been noted that 20-30% of tuberculous lymphadenitis may be complicated in such a way [13]. These patients manifest clinical or radiological deterioration of preexisting lesions and may even present with the development of new tuberculous lesions, after initiation of treatment, despite good therapeutic compliance [1]. This entity is more prevalent among HIV-positive patients but is also being more and more documented in HIV-negative patients, especially in cases of lymph node tuberculosis [13], as in our patient. The pathophysiological mechanism of PR development remains abstruse and has been postulated to pivot on the patient's immunological response. Specifically, PR has been associated with delayed immune activation, a finding corroborated by the observation that patients with PR may present with a negative tuberculin skin test (TST) and decreased lymphocyte blastogenesis initially, and a positive TST with increased lymphocyte blastogenesis after the initiation of treatment [14]. Furthermore, it has been found that in cases of extrapulmonary tuberculosis, a continuous increase in T-cell response may occur within a period of six months after initiation of treatment [15]. Interestingly, the patient had delivered eight months ago in the case we report. According to Cheng et al., approximately 90% of postpartum tuberculosis cases had extrapulmonary tuberculosis [16]. During pregnancy, Th2/Th3 responses (which lead to immunosuppression) are amplified, whereas Th1 response (which leads to proinflammatory responses) is inhibited in order for the maternal immune system not to reject the fetus. Since Th2 response has been implicated in the asymptomatic mycobacterial infection, the switch from Th2 towards Th1 response during the postpartum period may facilitate the development of disseminated lesions in the course of the immune reconstitution [17]. Although there are literature data that the Th2 cytokine predominance may return to nonpregnant Th1:Th2 ratios by four weeks postpartum [18], there are reported cases of immunity changes involvement within 4-5 months postpartum [17]. The time period from the initiation of anti-tuberculosis treatment to PR development varies and has been documented as three months, at most, for HIV-negative patients [19].

Therapeutical management of PR has raised controversy among authors. Close observation of the patient under anti-tuberculosis treatment, aspiration or excision of deteriorating lesions, as well as administration of anti-inflammatory agents or corticosteroids, comprise the treatment options. The utilization of monoclonal antibodies has also been proposed [20]. However, in cases of lymph node tuberculosis, data regarding immunotherapy, as well as corticosteroid therapy, do not suffice to convince [13]. Our patient continued receiving anti-tuberculosis treatment with concurrent administration of antibiotics, while CT-guided fluid drainage was also implemented for the psoas abscess. She demonstrated a favorable outcome during a follow-up period of one year.

## Conclusions

In summary, we presented a case where extrapulmonary tuberculous lesions deteriorated in an HIV-negative patient postpartum, who was not malnourished, more than three months after treatment initiation. Physicians should bear in mind that PR may develop during the postpartum period, possibly due to the shift from Th2 towards Th1 response, in the course of the immune reconstitution. To the authors' knowledge, the report of similar cases is only scarce. Awareness among medical doctors should be raised, as timely recognition of such tuberculosis complications and subsequent appropriate therapeutical approach are of crucial importance in order to ensure a favorable outcome.
